# Systematic review of menstrual health and hygiene in Nepal employing a social ecological model

**DOI:** 10.1186/s12978-022-01456-0

**Published:** 2022-06-30

**Authors:** Aditi Sharma, Jennifer S. McCall-Hosenfeld, Yendelela Cuffee

**Affiliations:** 1 Stanford University, Palo Alto, CA USA; 2 Department of Public Health Sciences, Pennsylvania State College of Medicine, Hershey, PA USA; 3 Department of Medicine, Pennsylvania State College of Medicine, Hershey, PA USA; 4 Epidemiology Program, University of Delaware, Newark, USA

**Keywords:** Menstrual health, Hygiene, Nepal

## Abstract

Menstrual health and hygiene are a major public health and social issues in Nepal. Due to inadequate infrastructure to provide education, healthcare, and communication as well as religious teachings, women and girls are excluded from participation in many activities of daily living and community activities during menstruation. Evidence based research addressing menstrual health and hygiene in Nepal is scares. The objective of this paper is to review the current state of knowledge on menstrual health and hygiene in Nepal through a socio-ecological perspective. This systematic review identifies knowledge gaps and targets for future research and interventions. Studies from Nepal that examined factors contributing to menstrual health and hygiene were identified through searches across six databases (Medline, CINAHL, Web of Science, PsychInfo, Nepal Journals Online and Kathmandu University Medical Journal) in January 2019. The SEM is a public health framework that describes how health is impacted at multiple levels including the individual, interpersonal, community, organizational and policy levels. Key themes were identified, and factors contributing to menstrual health and hygiene were categorized as per the level of socio-ecological model (SEM). After a comprehensive literature review, twenty peer-reviewed publications, published between 2003 and January 2019 were included in this review. Eighteen studies were descriptive and two were interventional. The main outcomes reported were reproductive health concerns and menstrual hygiene practices. Nine studies focused on knowledge, attitude, and practices regarding menstruation, seven studies highlighted reproductive health issues, three studies focused on prevalence of culturally restrictive practices, and one on school absenteeism and intimate partner violence. Lack of awareness regarding menstrual health and hygiene, inadequate WASH facilities, no sex education and culturally restrictive practices makes menstruation a challenge for Nepali women. These challenges have negative implications on women and girls’ reproductive as well as mental health and school attendance among adolescent girls. There are gaps in the evidence for high quality interventions to improve menstrual health and hygiene in Nepal. Future research and interventions should address needs identified at all levels of the SEM.

**Supplementary Information:**

The online version contains supplementary material available at 10.1186/s12978-022-01456-0.

## Plain language summary

Menstrual health and hygiene are major public health and social issues in Nepal, due to a lack of health and education infrastructure, gender-based disparities and restrictive cultural and social norms. This study reviewed the current state of research on menstrual health and hygiene in Nepal. The socio-ecological model (SEM)—a public health framework that examines individual, interpersonal, community, organizational, and policy impacts on health—was employed to describe impacts on menstrual health and hygiene. After a comprehensive literature review, 20 papers were included in this analysis, of which 18 were descriptive and two were interventional. The primary menstrual health and hygiene outcomes reported were reproductive health concerns and menstrual hygiene practices. Key themes identified were knowledge, attitude, and practices regarding menstruation, reproductive health issues, prevalence of culturally restrictive practices, school absenteeism and intimate partner violence. The study results indicated lower menstrual health and hygiene challenges arose from lack of education around menstruation, lack of proper water, sanitation, and hygiene (WASH) facilities, poor sex education, and culturally restrictive practices. These challenges negatively impact women and girls’ reproductive health as well as mental health and school attendance. There is need for high quality, effective interventions to improve menstrual health and hygiene in Nepal. Future research and interventions should address improvements in menstrual health and hygiene at all levels of the socio-ecological model.

**Supplementary Information:**

The online version contains supplementary material available at 10.1186/s12978-022-01456-0.

## Background

Menstruation is a natural physiological process that most women experience during their reproductive years [1]. The Joint Monitoring Program (JMP) of the World Health Organization (WHO) and the United Nations International Children's Emergency Fund (UNICEF) highlight the importance of managing menstruation hygienically and with dignity. In order to accomplish this, women and girls must have access to clean menstrual management materials, that can be changed as frequently as necessary, privacy, access to water and soap for washing, and access to appropriate disposal facilities [2]. In addition, women and girls’ require information on menstrual health and hygiene. For women and girls to live a healthy, productive and dignified life, effective menstrual health and hygiene is essential [3].

Menstrual hygiene management is an important health and social issue in Nepal. Nepal is a predominantly Hindu country [4]. Although it is not explicitly stated in any Hindu scriptures, many Hindus believe that menstruating women and girls are impure [4]. Due to religious beliefs about the impurity of women during menstruation, many women and girls are excluded from participation in typical daily and community activities during menstruation. Nine out of ten women and girls in Nepal report restrictions during menstruation including being prohibited from entering prayer rooms, temples and kitchens, touching their male family members, sleeping in their own bed, and going to school [4].

Nepal’s cultural and sociodemographic features contribute to practices that sustain inadequate menstrual hygiene such as lack of access to information regarding menstruation, safe water, clean, private toilets and menstrual products. Nepal’s poor economic standing contributes to low quality infrastructure in education, health, and communication [5]. In Nepal, gender discrimination is frequently exercised in the name of religious, cultural and social norms [5]. Women are often not involved in decision making for household matters and are uncomfortable challenge harmful cultural and religious practices [3].

Restrictions placed on women and girls during their menses are more likely to be practiced in rural and remote areas of Nepal. The most extreme of these practices is the ‘Chhaupadi Pratha’ (Chhaupadi), which is prevalent throughout the far western region and some parts of the mid-western region of Nepal [6]. Chhaupadi is a tradition in which women are banished to outdoor sheds (often where animals are kept) during their menses, and are considered impure and untouchable. Although the Chhaupadi tradition was legally abolished by the government of Nepal in 2005 and criminalized in 2017, it is still widely practiced in these areas, with menstruating women ostracized for about seven days every month [6]. Local residents believe that stopping this tradition would be offensive to the Gods and would bring bad luck and misfortune [7].

Literature pertaining to menstrual hygiene in Nepal is lacking [8]. Menstrual health and hygiene has not previously been examined to understand its impact at individual, interpersonal, community, organizational and policy levels.

### Social ecological model

The social ecological model (SEM) is a paradigm used to guide public health research and practice [9]. The model describes how individual and environmental characteristics influence health outcomes. Examining a public health problem using the SEM shows that health is impacted by factors on multiple levels including the individual, interpersonal, community, organizational and policy levels [10]. Thus, the SEM is a useful framework for examining menstrual health and hygiene in the developing world [11]. For example, on an individual level, women and girls may lack knowledge about menstruation. Poor knowledge on menstruation leads to misconceptions that perpetuate stigma and cultural restrictions as well as poor hygiene [3]. On an interpersonal level, they may face various restrictions during their menses due to cultural beliefs [12]. These cultural beliefs not only hinder proper menstrual health and hygiene but also lower the self-esteem of women and girls. On the policy level, there may be a lack of programs to promote menstrual health and hygiene such as ensuring water, sanitation and hygiene (WASH) in schools [11]. Lack of proper WASH facilities in schools lead to school absenteeism among menstruating adolescent girls [11]. Although most menstrual health and hygiene research and interventions have been focused on individual and interpersonal levels, exploration of factors affecting menstrual health and hygiene at all levels of the SEM is important [13].

Poor menstrual health and hygiene has gained global attention in recent years. Academics, non-governmental organizations (NGOs), the United Nations (UN) and other international agencies have called for an end to the gender gap in school education. Promoting menstrual health and hygiene is one promising means to achieve this goal, as menstruation contributes to this gender gap; girls face challenges to ful participating in school due to limitations regarding menstrual management in school environments [12].

The objectives of this paper are (1) to review the current state of knowledge on menstrual health and hygiene in Nepal by employing a socio-ecological perspective and (2) to understand the challenges, barriers and facilitators to optimizing menstrual health and hygiene.

## Methodology

A literature search was conducted on January, 2019 in five databases: Medline, CINAHL, Web of Science, Nepal Journals Online (NepJOL) and Kathmandu University Medical Journal (KUMJ). Due to limited literature on this topic, broad key terms were used: ‘menstruation’, ‘Chhaupadi’, ‘Chaupadi’ and ‘Nepal’. Key terms were combined through the Boolean operators (‘AND’ and ‘OR’). An example of the search strategy is as follows: (((((menstruation) OR chaupadi) OR chhaupadi)) AND Nepal). The inclusion and exclusion criteria were defined before the literature search and followed strictly. The inclusion criteria were: (1) papers that included women of reproductive age [15–49] in Nepal, and (2) studies that examined factors contributing to menstrual health and hygiene. Study designs included in the articles reviewed were qualitative, quantitative and mixed methods. Only papers published in English were included. Organizational reports, policy statements and training manuals were excluded from this review a priori.

The titles and abstracts of all papers identified during the literature search were reviewed to check relevance and appropriateness. Duplicates were removed manually. The reference lists of all identified articles were reviewed to identify additional manuscripts that may have been missed during the initial search. After the full review of the final list of all selected titles and abstracts, a quality appraisal was conducted for all the papers.

Quality assessment was conducted using the approach described by Banks et al. [14]. Each paper was given a quality score using the modified version of the Strengthening The Reporting of Observational Studies in Epidemiology (STROBE) and the Qualitative Research Review Guidelines (RATS) quality assessment tools [14]. The papers were assessed to have either high risk of bias, medium risk of bias, or low risk of bias, based on their study design, sampling methods, data collection, data analysis and interpretation. The studies were considered low risk of bias if most of the criteria in the quality assessment tool were met; medium risk of bias if some of the criteria were met; and high risk of bias if few or none of the criteria were met (Additional file 1).

Due to variation in the study designs of the papers included in this review we developed a data abstraction form to abstract important elements from each paper. The abstraction form included: the type of study, author(s), title, journal or publication, years published and conducted, research objectives, participants characteristics, study design, setting, sampling size and methods, data collection methods, ethical considerations, data analysis techniques, outcomes measured, key findings and study limitations.

We identified three key menstrual health and hygiene categories that were examined in the papers: reproductive health concerns, menstrual hygiene practices and mental health concerns. After identifying key themes from each paper, factors contributing to menstrual health and hygiene were categorized as per the level of SEM and described in detail. The interactions between levels in SEM were then examined. The SEM was adopted from the Maternal, Newborn, Child Health and Nutrition Guide Module on Social Ecological Model developed by UNICEF [15].

As noted above, there are five main levels of SEM: individual, interpersonal, community, organizational and policy/enabling environments. The individual level includes, knowledge and literacy, attitudes, values, goals, expectations, behaviors, self-efficacy, developmental history, gender, age, religion, race/ ethnicity and economic status [15]. The interpersonal level includes social networks, social support systems and customs and traditions [15]. The community level includes built environment, village associations, community leaders, local businesses and transportation [15]. The organizational level includes organizations and social institutions that affect how or how well services are provided to an individual or group [15]. Finally, the policy/enabling environment level includes local, state, national and global laws and policies [15].

Ethical approval of any kind was not required for this review as it carries minimum risks.

## Results

### Study selection

**Fig. 1:**
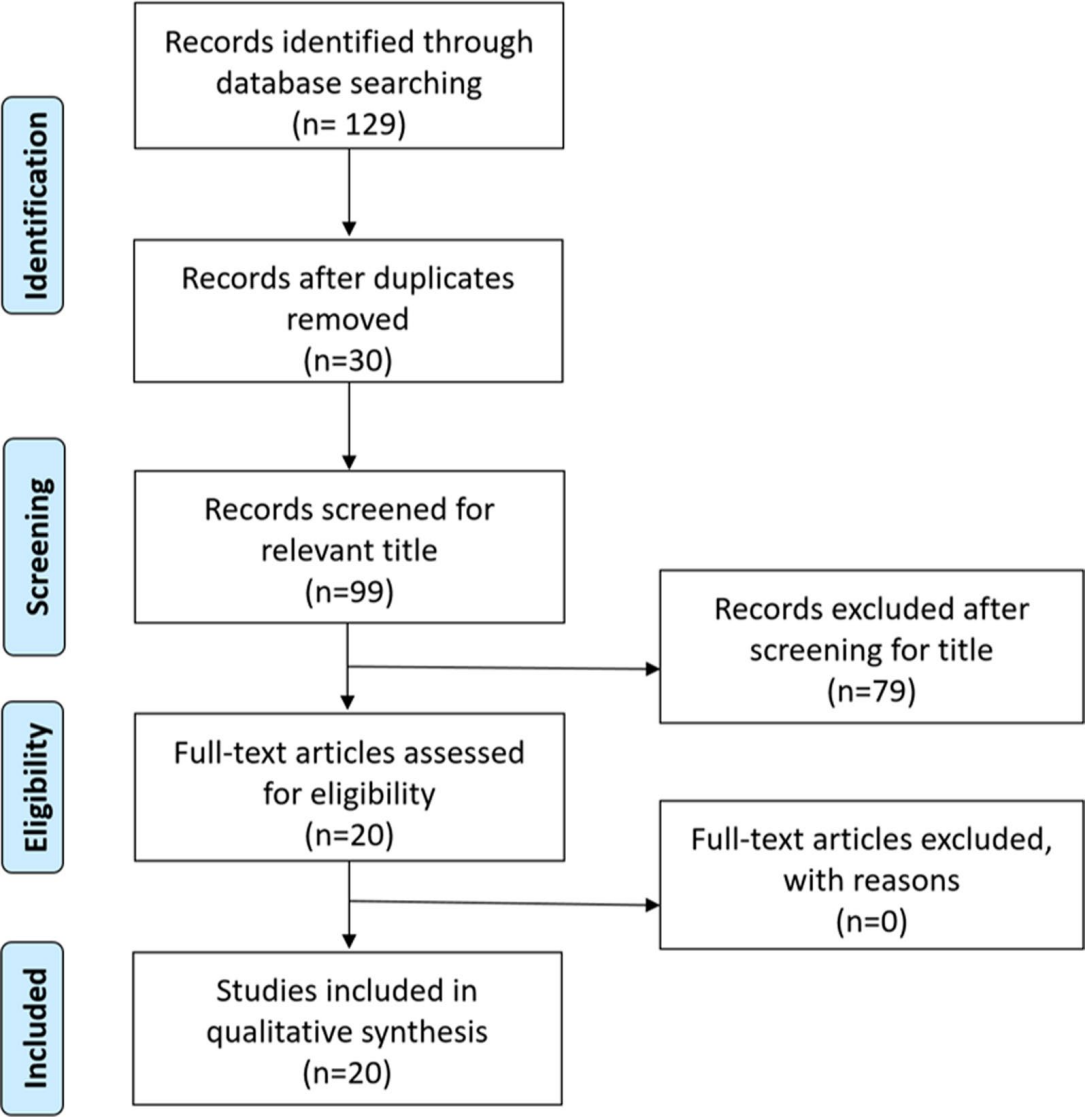
Search strategy with PRISMA flow diagram [16]

A total of 129 papers were identified through database searches. Thirty duplicates were found and removed. Ninety-nine papers were removed after screening titles and abstracts. Twenty papers were assessed for quality. Although the limitations and biases of all papers were examined, none of the papers were excluded from this study. A total of twenty papers were included in this literature review (Fig. 1).

### Study characteristics

**Fig. 2:**
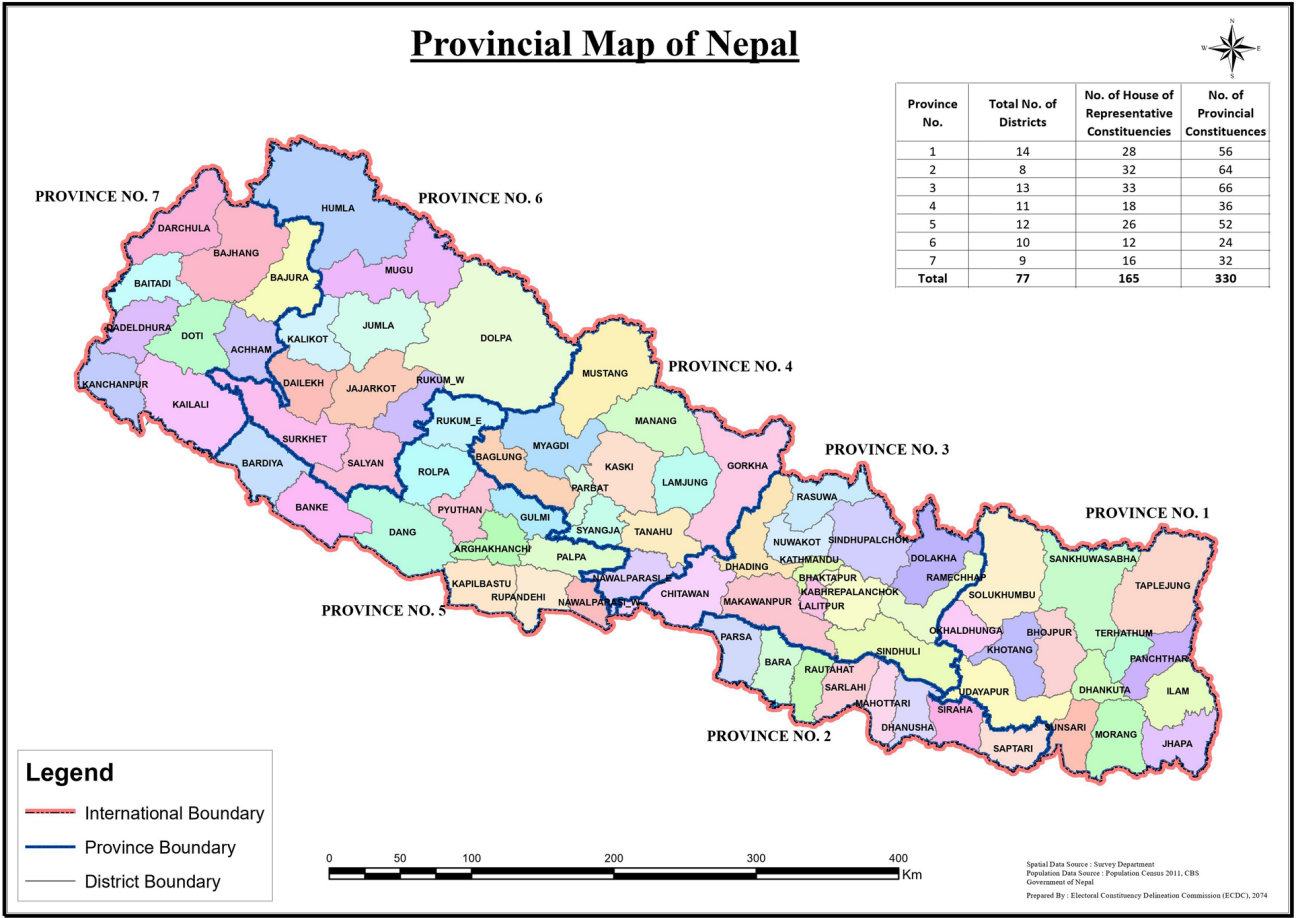
Provincial Map of Nepal [17]

The description of the studies included in this review have been summarized and presented in Table 1. These characteristics include study design, methods, location, year of publication, and primary outcomes assessed. Twenty studies that met inclusion criteria were identified. Studies were published between 2003 and 2018; most were published in 2016 and 2018. Studies were conducted in all of Nepal’s seven provinces except province Number Two; most studies were conducted in province Number Three, a map of the provinces is provided in Fig. 2. The federal provinces are divided on the basis of Nepal’s social and cultural diversity with the rationale of inclusive and equitable development and decentralization and devolution of autonomy.

**Table 1:** Study description

SN	Study	Journal	Purpose	Study design	Setting	Sample	Results
1	Buaman et al. 2019	Global Public Health	To explore the role of caste/ethnicity in menstrual knowledge, attitudes and practices in Nepal	Community-based, cross sectional	Doti, Dang, Kapilvastu, Lalitpur, Chitwan, Tanahun, Makwanpur, Saptari, and Jhapa districts	679 women	Caste/ethnicity was a significant predictor of menstrual knowledge and practices. The caste/ethnic groups Tarai/Madhesi/Other, Newar, Janajati, and Muslim all had statistically significant fewer odds of positive menstrual practices compared to Brahman/Chhetri (high caste groups), with Janajati (indigenous ethnic groups) having the poorest outcomes
2	Amatya 2018	PLoS One	To assess the prevalence of *Chhaupadi* practice among adolescent girls, observe the physical conditions and sanitation of the living spaces during *Chhaupadi*, assess the lived experience of *Chhaupadi* among adolescent girls and the perceptions of local stakeholders towards *Chhaupadi*	Mixed methods study	Accham district	107 adolescent girls	Majority of the girls practiced exile during menstruation. Around 4% were exiled to traditional Chhau sheds, 82% to livestock sheds and 11% to outside courtyards. Around 3% stayed inside the house but practiced some form of menstrual taboos. Only 30% of girls who stayed outdoors had toilet facilities Participants reported having various psychological problems, including loneliness and difficulty sleeping while practicing Chhaupadi. Notably high proportions of the living spaces lacked ventilation/windows, electricity and a warm blanket and mattress for sleeping. Three of the girls were physically abused; nine were bitten by a snake
3	Budhathoki 2018	BMC Women's Health	To describe the experiences and perceptions of women and adolescent girls on menstrual hygiene management in post-earthquake Nepal	Mixed methods study	Sindhupalchok district	127 women and girls living in temporary shelters	During a disaster, menstrual hygiene was rated as the sixth highest overall need and perceived as an immediate need by 18.8% of the respondents. Reusable sanitary cloth were used by about 66.7% of the respondents before the earthquake and remained a popular method (76.1%) post-earthquake. None of the respondents reported receiving menstrual adsorbents as relief materials in the first month following the earthquake. Women who were in the age group of 15–34 years, did not go to school, were married and previously used reusable sanitary cloth were more likely to use the reusable sanitary cloth
4	Cardoso 2018	BMJ Sexual Reproductive Health	To record the prevalence of menstrual restrictions experienced by married women and examine potential associations between IPV in the past year and menstrual restrictions imposed by husbands and/or in-laws among women in three districts of Nepal	Baseline data from a larger randomized control trial	Nawalparasi, Kapilvastu and Chitwan districts	1800 women	Nearly three out of four women (72.3%) reported experiencing high menstrual restriction, or two or more types of menstrual restriction. When controlling for demographic variables and IPV, no type of IPV was associated with high menstrual restrictions
5	Yadav 2018	Journal of Nepal Health Research Council	To assess the current knowledge, attitude and practice of school adolescents on menstrual hygiene management	Cross-sectional study	Doti district	276 students from grade seven and eight of 11 schools	Majority of the respondents had fair to good knowledge on menstrual hygiene management However, more than half the respondents did not engage in good menstrual hygiene practices. Around half of the respondents had positive attitude towards menstrual hygiene management related issues
6	Parajuli 2018	Bibechana	To find knowledge and practice on menstrual hygiene; and perspective of Chaupadi (menstrual shed) among the reproductive age group female	Community based mixed methods study	Pyuthan district	109 participants	Although majority of the respondents were aware of menstruation and menstrual hygiene, there was a gap in practice. Around 40% used sanitary pad during their menstrual flow but most (65.1%) of them did not dispose them; 16.5% bury in nearby ground and 18.4% burn. Different traditional practice followed were use of separate utensils, not allowed to see sun, restriction to- go outside, cook food, usual food intake, worship, eat with others, sleep in usual bedroom and touch male members. Most (94.5%) of them experienced Chhaupadi during their menarche
7	Rajbhandari et al. 2018	Nepal Medical Journal	To explore the existing knowledge and practices on menstrual hygiene among adolescents	Descriptive study	Bhaktapur district	168 adolescent girls	Majority of the respondents were aware of menstruation as physiological process and used commercially available sanitary pads. The primary source of information on menstruation was their mother. However, 35.1% of them reported that they had no prior knowledge on menarche The major reasons for school absenteeism were discomfort, lack of continuous water supply and shame or fear of staining
8	Parajuli 2016	Journal of Kathmandu Medical College	To assess the knowledge and practices regarding menstrual hygiene among adolescent girls	A descriptive study	Morang district	102 adolescent girls	It was found that majority (83.3%) of girls knew that menstruation is a physiological process. More than half of the respondents (53.9%) were taught about menstruation by their mother. Only 33.3% of the respondents used sanitary pad during menstruation. Adolescent girls had different type of restrictions during menstruation like not being allowed to cook food, visit holy places and sleep in own house during menarche
9	Katwal 2016	Kathmandu University Medical Journal	To assess the relationship between stress and dysmenorrhea amongst the Nepalese medical students	Cross-sectional descriptive study	Kavre district	184 female medical students	65% of participants considered medical education to be stressful. Around 67% of the participants experienced dysmenorrhea. Of them, 85% experienced increase in frequency and severity of dysmenorrhea after joining medical college. Of participants experiencing dysmenorrhea, 29.45% missed classes and 17.39% participants had positive family history of dysmenorrhea
10	Sharma 2016	BMC Women's Health	To determine menstrual pattern among adolescent girls	Cross sectional study	Kaski district	260 adolescent girls	Around 64.2% girls had irregular menstrual cycle and significant association was found between regularity of menstruation and ethnicity. Dysmenorrhea was reported by more than half of the girls and significant association was found between severity of dysmenorrhea with school absenteeism and treatment needed
11	Lui 2016	International Journal of Gynaecology and obstetrics	To describe findings from a validated survey examining access to care, contraceptive needs, access to surgical care, menstruation-related healthcare needs, and barriers to receiving reproductive health care in Nepal	Two‐part population‐based, cross‐sectional, cluster‐randomized survey	15 districts	876 female of reproductive age (12-50 years)	The most common form of sanitary products used were non-reusable sanitary pads and reusable towels. Urban residents and educated women had increased odds of using pads. Every year increase in age and non-motorized transport to a primary healthcare facility had decreased odds of using a pad. About one forth women reported dysmenorrhea
12	Ranabhat 2015	Asia Pacific Journal of Public Health	To determine the factors of reproductive health problems related to Chhaupadi	Cross-sectional study	Kailali and Bardiya districts	Women of menstrual age (N = 672)	One fifth of the households practiced Chhaupadi. Livelihood conditions, access to water facilities, food restrictions during menstruation and Chhaupadi stay were significantly associated with reproductive health problems in women such as burning micturition, abnormal vaginal discharge, itching in genital part, painful and foul smelling menstruation
13	Crawford 2014	Culture, Health and Sexuality	To understand menstrual stigma in the context of religiously-based menstrual restrictions imposed on women, we conducted qualitative research with women in Nepal	Qualitative study	Kathmandu district	Female students and NGO workers [Focus group 1 (n = 4), Focus group 2 (n = 4), Individual interviews (n = 11)]	Women reported being unprepared for menarche that then led to distress and stigmatization
14	Shrestha 2013	International Journal of Nursing Education	To assess the knowledge, attitude and practice of adolescent girls on menstrual hygiene	Interventional study	Kaski district	60 adolescent girls	Educational intervention showed significant increase in knowledge, attitude and practice towards menstrual hygiene
15	Sapkota 2013	Journal of Kathmandu Medical College	To assess the knowledge and practices regarding menstruation among school going adolescents	A descriptive study	Sunsari district	61 female adolescents	Dysmenorrhea was the commonest problem faced during menstruation (78.7%) followed by back pain and excessive blood loss. More than half of respondents (54.1%) used sanitary pads and frequency of changing pads twice a day was highest (50.8%). Initial reaction was of fear/apprehension at menarche by 36.1% of girls whereas 44.3% perceived it as an expectant process. Girls still faced different types of restrictions like not being allowed to visit holy places, not being allowed to cook and touch male family member etc
16	Paneru 2013	Asian Journal of Medical Sciences	To identify prevalence and factors associated with RTIs among married women of reproductive age	Cross-sectional study	Kaski district	282 participants	Prevalence of RTI symptoms was estimated to be 78.9 percent. Common reported symptoms were backache (71%), low abdominal pain (67%), watery vaginal discharge (56%), genital itching (51%), burning urination (44%) and curdy discharge per vagina (26%). Illiterates, those who had sexual contacts during menstrual periods and those who do not clean genitalia after sexual act were significantly more at risk (OR = 5.35,8.33 and 3.11) of having RTIs than those who do not have these attributes
17	Oster 2011	American Economic Journal—applied economics	To evaluate the causal impact of providing modern sanitary products on girls’ schooling	Randomized controlled trial	Chitwan	198 (99 control, 99treatment)	Menstruation has a very small impact on school attendance. Girls miss an estimated total of 0.4 days in a 180 day school year. Improved sanitary technology has no effect on reducing this (small) gap: girls who randomly received sanitary products were no less likely to miss school during their period
18	Pramanik 2010	Journal of Institute of Medicine	To assess the relationship between the degree of stress and incidence of dysmenorrhea amongst the young Nepalese medical students	Prospective study	Kathmandu district	Young, unmarried, non-smoker female medical undergraduate students (age: 18-20 years, n = 104) having no pelvic pathology	Result indicated that the stress score is significantly higher (31.30 vs. 18.81) in women suffering from dysmenorrhea compared to women with normal menstruation
19	Adhikari 2007	Kathmandu University Medical Journal	To evaluate the knowledge and practice on different aspects of menstrual hygiene among adolescent girls	Cross sectional study	Chitwan district	150 young girls	Only 6.0% of girls knew that menstruation is a physiologic process, 36.7% knew that it is caused by hormones. Majority of them used disposable pads but were not aware of proper disposal practices
20	Padhye 2003	Kathmandu University Medical Journal	To find out the incidence of Menstrual Morbidity and their mode of presentation	Prospective study	Kathmandu district	525 female patients with menstrual problems	Menstrual morbidity was found to be 43.75%. More than 90% of women followed traditional rituals during menarche and more than 75% followed discriminating traditional rituals during every cycle

Fifteen studies were quantitative; one study was qualitative and three were mixed methods. The majority (n = 18) of the studies were descriptive and only two were intervention studies. Of the twenty studies, nine focused on knowledge, attitude and practices regarding menstruation, seven focused on reproductive health issues associated with menstruation, three on prevalence and experiences with cultural rituals and restrictive practices during menstruation such as Chhaupadi, one on school absenteeism and one on intimate partner violence. The study characteristics are shown in Table 2.

**Table 2:** Study characteristics (n = 20)

Characteristics	Frequency	%
Design		
Descriptive	18	90
Intervention	2	10
Method		
Quantitative	15	75
Qualitative	1	5
Mixed methods	4	20
Location		
Province 1	2	10
Province 2	0	
Province 3	9	45
Province 4	3	15
Province 5	2	10
Province 6	1	5
Province 7	3	15
Year of publication		
2003–2012	5	25
2013–2018	15	75
Primary outcomes		
Reproductive health concerns	9	45
Menstrual hygiene practices	12	60
Mental health concerns	5	25

As per the quality assessment, six studies were identified as having low risk of bias, twelve as medium risk of bias and one as high risk of bias. Potential biases included response rates not reported, potential confounders not taken into consideration, study samples not representative of the broader population, and tests for statistical significance that were not undertaken. Due to limited studies in menstrual health and hygiene in Nepal, we included all the studies regardless of their risk of bias.

### Outcomes of menstrual health and hygiene

The three main menstrual health and hygiene outcomes reported in the papers were reproductive health concerns, menstrual hygiene practices and mental health concerns. *Reproductive health concerns* reported included menstrual cycle irregularities, dysmenorrhea and other genitourinary complaints such as burning during micturition, abnormal discharge, genital itching, pain, and foul smelling menstruation [8, 18–25].

*Menstrual hygiene practices* described by the papers included use of sanitary products such as commercial pads, homemade reusable pads and menstrual cups, bathing and cleaning perineal areas during menstruation, frequency of changing menstrual pads during the day, washing hands after changing pads, washing and drying of reusable menstrual pads and various restrictions faced during menstruation [6, 19, 23, 26–34].

*Mental health concerns* described by the studies encompassed confusion, stress, shame, pain, fear of leakage and teasing [18, 25, 27, 33, 35].

### Factors that impact menstrual health and hygiene by each level of SEM

#### Individual level

On an individual level, factors that impacted menstrual health and hygiene were demographics (age, education, residence, economic status, and marital status), knowledge of menstruation, and negative experiences related to menstruation.

Six papers reported on age in association with menarche, dysmenorrhea, irregular menstrual bleeding and sanitary pad use [18, 23, 24, 30, 32, 34]. Liu et al. (2016) reported that the median age for women with dysmenorrhea was 26 years and with irregular menstrual bleeding was 34 years [19]. They also reported that every year increase in age led to decreased odds of using commercial pads compared to reusable towels [19]. Sharma et al. (2016) found no statistical significant association between menstrual irregularity and age [24]. Amatya et al. (2018) did not find any statistically significant effect of age on the practice of menstrual exile [6].

Four studies reported on educational factors including types of school, level of education and school absenteeism [19, 20, 24, 31]. Sharma et al. (2016) found no statistically significant association between menstrual irregularity and type of school (private vs. government). Liu et al. (2016) stated that women who were educated had an increased likelihood of using commercial pads than those who were not educated. Findings from Sharma et al. (2016) showed that school absenteeism was significantly associated with the severity of dysmenorrhea. Another cause for school absenteeism reported by Rajbhandari et al. (2018) was fear of leakage and staining during menstruation. Oster et al. (2011) found no significant effect of provision of menstrual cups on girls’ school attendance. The study reported that 43.8% of the girls’ listed period cramps as a reason for school absenteeism during menstruation.

Residence impacted several aspects of menstrual health and hygiene [8, 19]. Ranabhat et al. (2015) reported that people living in Kailali district in Province Number Seven as opposed to Bardiya district in Province Number Five had significantly higher reproductive health problems such as burning during micturition, abnormal discharge, genital itching, or painful and foul smelling menstruation [8]. The two districts are adjacent and both have migrants from places where Chhaupadi is practiced [8]. Liu et al. (2016) stated that women who were from urban areas had an increased likelihood of using commercial pads compared to rural women [19].

Two studies reported findings on the impact of women’s economic status on menstrual health and hygiene [6, 8]. Women with poor economic status had significantly higher reproductive health problems than more economically advantaged women [8]. In one study, economic status did not have any statistically significant difference among people who practiced menstrual exile and those who did not [6].

Marital status was reported only by Amatya et al. (2018) in association with menstrual health and hygiene. However, they did not find any statistically significant difference in marital status among people who practiced menstrual exile and those who did not.

Eight papers reported on knowledge of menstruation, including understanding the physiology of menstruation, knowledge of menstrual hygiene practices such as use of sanitary pads, frequency in changing pads, bathing and cleaning genital areas during menstruation, and appropriate disposal of used sanitary pads [23, 29–35]. Rajbhandari et al. (2018) reported that one third of their study participants had no knowledge of menstruation before menarche [31]. Overall, the studies that reported knowledge on menstruation reported poor or insufficient knowledge of menstruation and menstrual hygiene among participants [23, 29–34]. Parajuli et al. (2016) recommended conducting meta-analyses and a systematic review on studies conducted on knowledge about menstruation in different parts of Nepal.

Five papers reported on negative experiences related to menstruation [18, 25, 27, 33, 35]. Two studies stated that women and girls perceived menarche as confusing, stressful and inconvenient [27, 35]. Similarly, Yadav et al. (2017) reported that menstruation was a source of shame, pain, and fear of leakage and odor as well as teasing from boys [33]. Dysmenorrhea and stress were shown to be positively correlated in studies conducted by Pramanik et al. (2010) and Katwal et al. (2016) [18, 25].

#### Interpersonal level

Factors identified on the interpersonal level affecting menstrual health and hygiene included ethnicity, education level of husbands, family members as source information about menstruation, culturally restrictive practices, and intimate partner violence.

Three papers reported on ethnicity and its association with menstrual health and hygiene [8, 24, 26]. Sharma et al. (2016) reported a statistically significant difference on menstrual cycle regularity by ethnicity [24] with Hill ethnic groups more likely to have irregular menstrual cycles than the Dalits and other ethnic groups [24]. The study called for further research on disparities among ethnic groups regarding menstrual regularity [24]. On the contrary, Ranabhat et al. (2015) found that women whose ethnicity was upper caste had significantly higher reproductive health problems compared to lower caste women. [8]. Similarly, Cardosa et al. (2019) reported that women and their husbands who came from upper castes reported higher levels of menstrual restrictions [26].

Three studies reported that women and girls mostly received information on menstruation from their mothers and friends [23, 30, 35]. The information received included restrictions and impurity associated with menstruation, cramps, how to sit to avoid stains, and how to fold and clean menstrual cloths. Girls received conflicting information from mothers, teachers and friends regarding menstruation [35]. In one study, the majority of the girls received information regarding menstruation from their teachers. Teachers taught girls about menstruation when teaching about sexually transmitted diseases, infections and cervical cancer [35].

Thirteen studies reported on various culturally restrictive practices faced by women and girls during menstruation [6, 8, 20, 21, 23, 26, 27, 29, 30, 32–35]. Restrictions include abstaining from going to the kitchen, prayer rooms and temples, eating certain foods, taking a bath and touching men including their husbands, not attending school and living outside the house in seclusion (i.e. Chhaupadi) [6, 8, 20, 21, 23, 26, 27, 29, 30, 32–35]. Culturally restrictive practices were followed due to various superstitions. It was believed that menstruating girls would curse their house with just a look [35]. Girls also believed that they would fail their exams, fall sick or become infertile if they prayed during their menstruation [35]. Women and girls believed that refraining from these restrictions would anger the Gods and bring bad luck to their families [6, 33]. Reproductive health problems were significantly higher among women who faced food, water and bathing restrictions or stayed in Chhaupadi sheds compared to those who did not [8].

Of note, Cardosa et al. (2019) found that high menstrual restrictions were not significantly associated with intimate partner violence (IPV). The study recommended further research to assess menstrual restrictions and their potential association with IPV. In contrast, Amatya et al. (2018) reported physical abuse during Chhaupadi [6].

#### Community level

Findings on the community level were sparse. Two studies reported on community level factors affecting menstrual health and hygiene. Budhathoki et al. (2018) examined menstruation-related disaster management after a massive earthquake in 2015 and found that women and girls did not receive any menstrual hygiene products as relief items within the first month after the earthquake [28]. Parajuli et al. (2016) reported that mothers’ groups were instrumental advocates against Chhaupadi in their communities [30].

#### Organizational level

Organizational factors related to menstrual health and hygiene that were reported included the educational system, WASH facilities in schools, information on menstruation through mass media, the role of NGOs in providing menstruation hygiene management education and training, and access to transportation.

Three studies reported on the need for improved sex education [26, 33, 34]. Women in the Crawford et al. (2014) study report a need for better sex education for youth prior to menarche [27]. The study recommended sexuality education interventions tailored to reduce stigma, increase women and girls’ agency in rejecting harmful traditions associated with menstruation, and normalize menstruation in a positive manner [27]. According to Yadav et al., (2017) 47.5% girls learned about menstruation in schools [33]. Ninety-eight percent of the girls in the Adhikari et al. (2007) study stated that they were not adequately informed about menstruation from their education at school. The study recommended motivating teachers and health workers to improve their knowledge on menstrual health and hygiene in order to help adolescent girls [34].

Three studies reported on inadequate WASH facilities in schools [31, 33, 35]. Out of eleven schools observed by Yadav et al. (2017), four had separate toilets for males and females, three had running water available, one had soap available in the toilet and four had open defecation [33]. Rajbhandari et al. (2018) reported lack of continuous water supply, lack of private space and lack of separate bathrooms for girls in schools as reasons for school absenteeism among girls during their menses [31]. Morrison et al. (2018) reported that none of the schools in their study had private washing and drying facilities [35]. Only three out of twelve schools had waste disposal facilities [35].

Two studies reported on mass media as a source of information for women and girls regarding menstrual health and hygiene [6, 27, 31, 33]. Yadav et al. (2017) reported that 70% of the girls received information on menstruation from the local radio station [33]. Crawford et al. (2014) recommended using mass media campaigns to celebrate menstruation in a positive light to reduce societal stigma [27].

Five studies reported that NGOs played a major role in providing menstrual hygiene education and training [6, 28, 30, 33, 35]. According to Parajuli et al. (2018), Chhaupadi practice was decreasing due to awareness raising, social inclusion and advocacy by many organizations [30]. Yadav et al. (2017) described a promising practice regarding training to create sanitary pads. 28.3% of the female respondents had participated in a sanitary pad making training and the majority of them made their own pads after participating in those trainings [33]. Morrison et al. (2018) stated that girls, mothers and teachers felt optimistic about NGO workers providing information on menstrual management [35]. Amatya et al. (2018) recommended that NGOs develop and implement appropriate sex education programs in the community, especially in the far western region of Nepal, to address Chhaupadi [6]. In August 2017, the Government of Nepal passed a criminal code criminalizing Chhaupadi. Amatya et al. (2018) suggested temporary measures to protect women practicing Chhaupadi, since the law criminalizing Chhaupadi will take time to implement. Budathoki et al. (2018) recommended that organizations working in disaster relief should work to use culturally appropriate, local materials to make reusable sanitary towels [28].

Only one study reported on the role of access to transportation in menstrual health and hygiene [19]. Liu et al. (2016) reported that using non-motorized transportation to a primary care center was associated with decreased odds of using commercial sanitary pads as opposed to reusable towels in comparison to using motorized transportation to primary care center [19].

#### Policy level

There were no papers that described the impact of a policy change on menstrual health and hygiene. Three papers described policy recommendations based on their findings [8, 26, 34]. Ranabhat et al. (2015) recommended that Nepal’s Ministry of Health and Population should formulate policies to eliminate harmful practices related to menstruation that pose a threat to women’s health [8]. Adhikari et al. (2007) recommended that the government of Nepal should promote menstrual health and hygiene through different media programs, including television, radio channels and newspapers [34]. Integration of menstrual hygiene management efforts with the sustainable development goals (SDGs) was recommended by Cardosa et al. (2019) [26]. The SDGs are global goals set by United Nations Development Programme (UNDP) that recommend all governments to prioritize ending poverty, protecting the planet, and ensuring peace and prosperity for all.

## Discussion

**Fig. 3:**
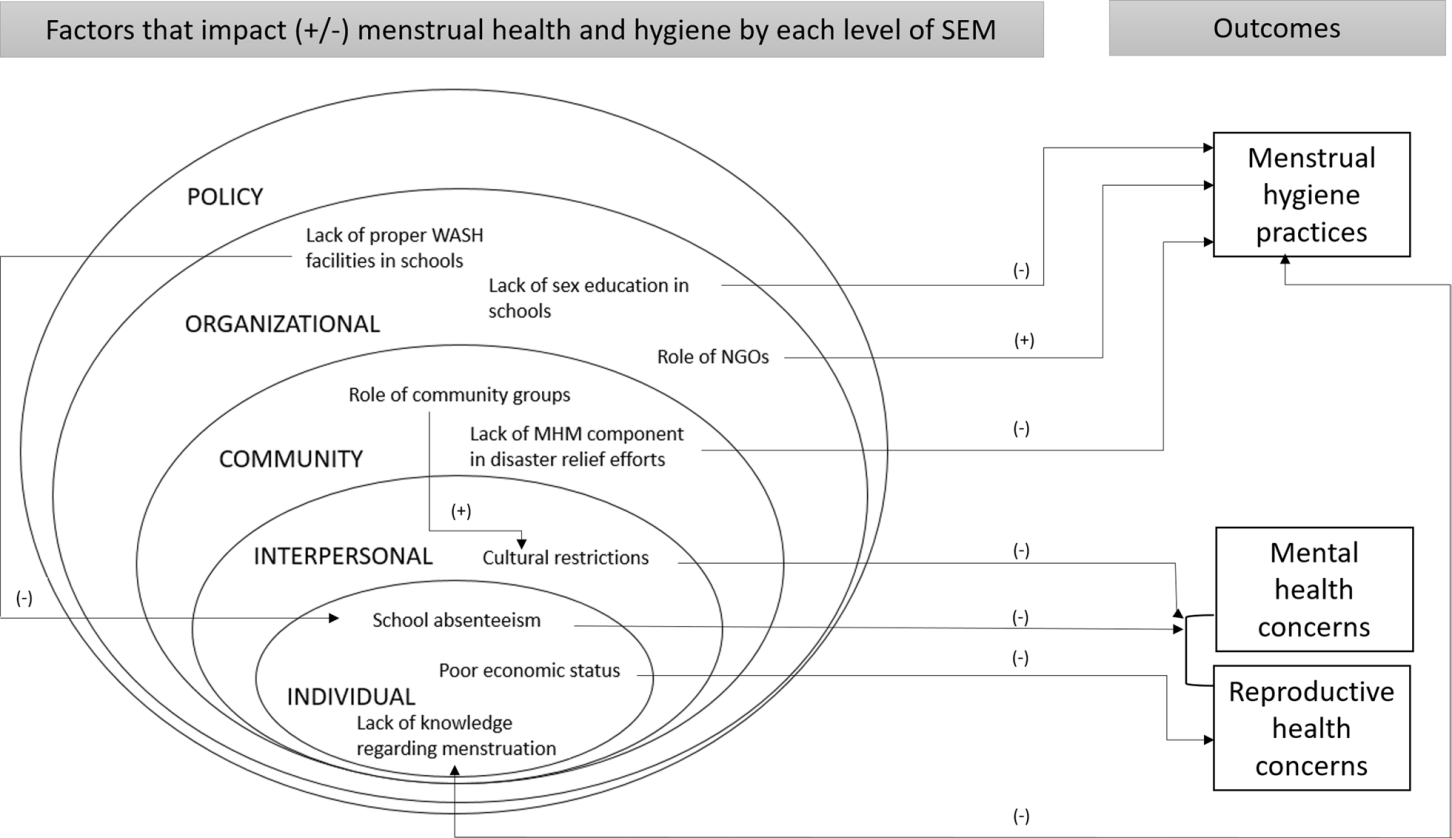
Factors that impact menstrual health and hygiene by each level of SEM

This systematic review examined studies of menstrual health and hygiene practices in Nepal. Twenty studies met our review criteria. Studies differed in quality and outcomes reported. Although all the studies included in this review were published in peer reviewed journals, the majority of them held medium risk of bias. Bias was often noted in studies that were published in national journals. Most of the studies focused on understanding the knowledge, attitude and practices regarding menstruation (twelve studies) reproductive health issues related to menstruation (nine studies) and mental health concerns (five studies). Although the majority of the studies explored culturally restrictive practices related to menstruation, only three papers specifically focused on it. One study focused on school absenteeism due to menstruation and one on the impact of culturally restrictive practices during menstruation on intimate partner violence. Figure 3 summarizes the key findings of this review with factors that negatively or positively impact menstrual health and hygiene by each level of SEM and their association with menstrual health and hygiene outcomes.

### Individual level

As per the review, the primary reasons for school absenteeism during menstruation were dysmenorrhea, fear of menstrual leakage and staining, and lack of WASH facilities in school. One study in the review found no association between school attendance and provision of menstrual cups. However, this study was conducted in an urban area, within a district (Chitwan), which has the sixth highest literacy rate in Nepal. Because only about 20% of Nepal’s population lives in urban areas, this may not reflect an intervention that is accessible to most of the women and girls in Nepal. Moreover, the gender gap in education is more pronounced in rural areas, suggesting that the results of this study are not generalizable to Nepal, especially the rural areas. More research is needed to understand the relationship between menstrual hygiene and school absenteeism. This review affirms the findings in other international literature that women and girls with low economic status are less likely to be aware of proper menstrual health and hygiene practices as well as lack access and affordability of menstrual hygiene products [36]. A study from Turkey showed that higher socioeconomic status was associated with positive menstrual experiences [37].

Lack of knowledge on menstruation was an important finding. Some studies associated this lack of knowledge with poor menstrual hygiene practices. This finding was in line with studies from other developing countries like India, Pakistan, China, Nigeria and Uganda [38–42]. The studies in this review reported negative experiences related to menstruation such as stress, confusion, inconvenience, as well as shame and teasing in school. This finding is similar to other studies in the developing world. For instance, studies in India and Kenya have found that girls perceived menarche as shocking, scary and shameful [43, 44]. Other studies based in India and Kenya reported some girls associated menstruation with maturity and expressed pride at starting menstruation [44, 45]. There is a need to positively reframe perceptions of menstruation through access to stigma-free factual information about menstruation [46].

### Interpersonal level

Another finding from this review comes from two studies that report women who come from upper castes were more likely to practice menstrual restrictions and had higher reproductive health problems than their lower caste counterparts [8, 26]. However, a study that was published after this review’s search process investigates the impact of caste and ethnicity on menstrual hygiene in Nepal, finding that women from upper castes had higher odds of positive menstrual practices compared to other castes [47]. The study reported that caste was a significant predictor of menstrual knowledge [47]. Definitions of caste and ethnicity varied among the studies. Thus, the impact of caste on menstrual health and hygiene is an area for future study.

The studies in this review found that family members were often primary sources of information on menstruation. However, the studies do not determine if the information received was accurate or affirming. Although the studies from this review do not directly address this, studies from India and Tanzania examining mothers and sisters as the main source of information on menstruation showed that information was provided after menarche, rather than before, and that this information was guided by their misconceptions. Because family members are the main information providers, there is also a need to equip mothers and other family members with accurate information on menstrual health and hygiene [41, 48].

Cultural restrictions were reported in twelve out of the nineteen studies included in this review. Studies conducted in other countries in South Asia and Africa such as India, Bangladesh, Iran, Kenya and Ethiopia also practiced similar cultural restrictions that stem from misconceptions associating menstruation with impurity [49–53]. Since these practices are deeply rooted in culture, a study in Bangladesh found no significant change in these practices even after educational interventions [54]. These studies highlight the need for interventions to account for cultural and religious imperatives.

### Community level

A study in this review reported that after the devastating earthquake in Nepal in 2015, immediate emergency relief items did not include menstrual hygiene products [30]. Globally, as the number of displaced people is increasing, menstrual hygiene still remains a neglected aspect of hygiene interventions in emergency situations [55]. There is a need for humanitarian agencies to plan relief missions in a gender-sensitive and inclusive manner [55]. Menstrual hygiene supplies are a basic human need and must be viewed as such.

The role of mothers’ groups in advocating against Chhaupadi and promoting menstrual hygiene was highlighted by a study in this review [30]. Most villages in Nepal have mothers’ groups who come together to talk about health, nutrition and hygiene related issues. We recommend utilizing existing organizations such as these mothers’ groups to promote quality information on menstrual health and hygiene.

### Organizational level

Several studies in this review highlighted the need for sex education to fight stigma associated with menstruation, empower women and girls to understand their bodies and help the community to normalize menstruation [6, 27, 34]. Providing effective sex education in school improves healthy sexual maturation and hygiene [56]. Menstruation is a topic that falls underneath the broader category of sexuality issues; thus; in order to change knowledge, attitudes and beliefs regarding menstruation, comprehensive sex education is essential [57].

Poor WASH facilities in schools were reported in this review. Inadequate WASH facilities in schools not only compromises girls’ ability to maintain menstrual hygiene but also their opportunity to obtain proper education [58]. Although UNICEF introduced WASH for Schools (WinS) in Nepal, there is a need for gender-specific toilet facilities, provision of sanitary materials, private changing areas and places for hygienic disposal of sanitary products in schools [59].

This review found that NGO’s played a major role in providing menstrual hygiene education and training. NGOs work in both implementation and advocacy roles to promote menstrual hygiene management [12]. Globally, NGOs have a vital role in raising awareness on menstruation and prompted government action regarding menstrual hygiene management [12].

### Policy level

At the time of this review, there were no studies that specifically evaluated policy interventions designed to effect menstrual health and hygiene conducted in Nepal. In 2017, Populations Services International Nepal conducted a scoping review and preliminary mapping of menstrual health and hygiene management in Nepal [60]. However, this report was created for an organization and was not subject to a peer review and dissemination in an academic setting. The report stated that policy documents in Nepal have failed to prioritize menstrual health and hygiene. Menstruation has only been incorporated within larger frameworks of adolescent sexual and reproductive health and water, sanitation and hygiene programs. Policy documents that include menstruation in Nepal are draft versions that have not been endorsed by the Government of Nepal [60]. Our recommendations are that the Government of Nepal should prioritize and incorporate menstrual health and hygiene policies under various sectors and call for multi-sectoral coordination.

### Interactions among SEM levels

There were two significant interactions noted between the SEM levels observed in this review. The community mothers’ group (community level) played an important role in advocating against culturally restrictive menstrual practices such as Chhaupadi (interpersonal level). Another interaction was the negative impact of poor WASH facilities in schools (organization level) on school absenteeism (individual level).

### Strengths and limitations

This systematic review provides a summary and critical appraisal of studies that have been conducted on menstrual health and hygiene in Nepal to date. To the best of our knowledge, this is the first systematic review on menstrual health and hygiene in Nepal. The findings of this study were organized based on the social ecological model providing a multilevel perspective on menstrual health and hygiene. This study also provides useful insights to the menstrual health and hygiene practices among women in Nepal.

The studies in this review were limited to the English language, hence we may have missed some relevant studies published in Nepali. The studies were heterogeneous with varied outcome measures. Therefore, an outcome assessment and meta-analysis was not possible for this review. In addition, one of the studies included in our research had high potential for bias. However, studies with high and low bias potential did not have conflicting findings.

### Implications for research

The lack of attention received by menstrual health and hygiene in Nepal is reflected by the limited numbers of high-quality studies gathered for this review. There are a growing number of grassroots initiatives to improve menstrual health and hygiene in Nepal. While this work is promising, rigorous studies that comprehensively evaluate interventions on menstrual health and hygiene are needed. Studies were largely descriptive; intervention studies and trials focusing on the efficacy or potential harm of menstrual health and hygiene interventional programs were limited. More research is needed to understand the relationship between menstrual hygiene and school absenteeism. The majority of the studies on menstrual health and hygiene have focused on adolescent girls; however, there is a critical need for studies that include adult menstruators. Current research studies do not include with individuals with disabilities, non-binary and transgender people and menstruators from other marginalized backgrounds. Importantly, future studies must incorporate culturally-appropriate interventions. Additionally, our finding that studies demonstrated interactions between different levels of the SEM reinforces the importance of providing multi-level interventions to improve menstrual health and hygiene. Finally, the need for well-designed studies that examine the impact of policy on menstrual health and hygiene is paramount. The findings of this review can be used to change policies and practice and provide recommendations for promoting menstrual health and hygiene.

## Conclusion

Menstruation is a challenge for women in Nepal due to lack of awareness regarding menstrual health and hygiene, lack of proper WASH facilities and sex education in schools, and culturally restrictive practices. This has negative implications on women and girls’ reproductive health, mental health, and ability to participate in education. There is a gap in the evidence for policy evaluations and high-quality randomized interventions that serve as evidence for efficacious interventions on menstrual health and hygiene in Nepal.

## Supplementary Information

**Additional file 1:** Study description.

## Data Availability

Not applicable.

## Abbreviations

SEM: Social ecological model
WASH: Water, sanitation and hygiene
NGOs: Non-governmental organizations
UN: The United Nations
NepJOL: Nepal Journals Online
KUMJ: Kathmandu University Medical Journal
STROBE: Strengthening the Reporting of Observational Studies in Epidemiology
RATS: Qualitative Research Review Guidelines
IPV: Intimate partner violence
UNDP: United Nations Development Programme
