# Supplementary material for: Systematic review of menstrual health and hygiene in Nepal employing a social ecological model
Source: Reprod Health. 2022 Jun 30;19:154. doi: 10.1186/s12978-022-01456-0 (PMC9245245; doi:10.1186/s12978-022-01456-0)
Supplement: Supplementary file 1 — Additional file 1: Study description. [file 12978_2022_1456_MOESM1_ESM.pdf]

Quantitative studies	Ranabhat et al 2015	Crawford et al, 2014	Yadav et al, 2017	Liu et al, 2015	Adhikari et al, 2007	Budhathoki et al, 2018	Sharma et al, 2016	Cardoso et al, 2018	Amatya et al, 2018	Padhye et al, 2003	Shrestha et al, 2013	Oster et al, 2011	Parajuli et al, 2018	Sapkota et al, 2013	Parajuli et al, 2016	Pramanik et al, 2010	Paneru et al, 2012	Katwal et al, 2016	Rajbhandari et al , 2018	Bauman et al, 2019
1. Sampling methods																				
1.1 Was the sample representative of the broader population?	1	N/A	1	1	2	1	1	1	1	2	2	1	1	1	2	2	1	1	1	1
1.2 Was recruitment of participants appropriate to the study question?	1	N/A	1	1	1	1	1	1	1	1	1	1	1	1	1	1	1	1	1	1
1.3 Adequate sample size (>100 or sample size calculation undertaken)	1	N/A	1	2	2	1	1	1	1	1	3	1	1	2	1	1	1	1	1	1
1.4 Response rate reported and acceptable (≥70%)	3	N/A	3	1	3	1	3	3	1	3	3	3	3	3	3	3	3	3	1	3
1.5 Control group is appropriate, clearly defined (if applicable)	N/A	N/A	N/A	N/A	N/A	N/A	N/A	N/A	N/A	N/A	N/A	1	N/A	N/A	N/A	N/A	N/A	N/A	N/A	N/A
2. Data collection																				
2.1 Sample characteristics clearly described	1	N/A	2	1	1	1	1	1	1	1	1	1	1	1	1	1	1	1	1	1
2.2 Means of collecting data (e.g. assessment tool, questionnaire, etc) valid, reliable	1	N/A	1	1	2	1	1	1	1	1	1	1	2	2	1	1	2	2	1	1
3. Data analysis / interpretation																				
3.1 Potential confounders taken into account during the analysis and interpretation	3	N/A	3	1	3	3	3	1	3	3	3	3	3	3	3	3	3	3	3	2
3.2 Tests for statistical significance undertaken, presented	1	N/A	3	1	3	1	1	1	1	3	1	1	3	3	3	3	1	1	1	1
**Qualitative studies (adapted from RATS)**																				
1. Study design																				
1.1 Study design is appropriate to the research question	N/A	1		N/A	N/A	1	N/A	N/A	1	N/A		N/A	1	N/A	N/A	N/A	N/A	N/A	N/A	N/A
1.1.2 Could a quantitative approach have worked better?	N/A	1		N/A	N/A	1	N/A	N/A	1	N/A		N/A	1	N/A	N/A	N/A	N/A	N/A	N/A	N/A

1.1.3 Justified why a particular method was chosen, e.g.: a)Interviews: experience, perceptions, behaviour, practice; b) Focus groups: group dynamics, convenience, non - sensitive topics; c) Ethnography: culture, organizational behaviour, interaction	N/A	3		N/A	N/A	1	N/A	N/A	1	N/A		N/A	2	N/A	N/A	N/A	N/A	N/A	N/A	N/A
2. Sampling methods																				
2.1 Criteria for selecting study sample is appropriate, e.g. purposive (diversity of opinion), random (generalizable to broader population), volunteer (hard to reach groups)	N/A	1	N/A	N/A	N/A	1	N/A	N/A	1	N/A		N/A	1	N/A	N/A	N/A	N/A	N/A	N/A	N/A
2.2 Details given of how recruitment was conducted and by whom	N/A	1	N/A	N/A	N/A	3	N/A	N/A	1	N/A		N/A	1	N/A	N/A	N/A	N/A	N/A	N/A	N/A
2.3 Details given on who chose not to participate and why	N/A	3	N/A	N/A	N/A	3	N/A	N/A	1	N/A		N/A	3	N/A	N/A	N/A	N/A	N/A	N/A	N/A
3. Data collection																				
3.1 Collection of data is comprehensive and appropriate. E.g. a) Was the study setting appropriate? E.g. protection of confidentiality for sensitive discussions; b) Is the role of the researcher(s) appropriate? How might they bias the study and results? e.g. Do researchers occupy dual roles (clinician and researcher	N/A	1	N/A	N/A	N/A	1	N/A	N/A	1	N/A		N/A	3	N/A	N/A	N/A	N/A	N/A	N/A	N/A

4. Data analysis/interpretation																				
4.1 Are interpretations clearly presented and supported adequately by evidence?	N/A	1	N/A	N/A	N/A	1	N/A	N/A	1	N/A		N/A	3	N/A	N/A	N/A	N/A	N/A	N/A	N/A
4.2 Indicators of quality																				
Descriptions of how themese were derived from the data (inductive or dedcutive)	N/A	1	N/A	N/A	N/A	1	N/A	N/A	1	N/A		N/A	3	N/A	N/A	N/A	N/A	N/A	N/A	N/A
Semi quantification when appropriate	N/A	3	N/A	N/A	N/A	3	N/A	N/A	3	N/A		N/A	3	N/A	N/A	N/A	N/A	N/A	N/A	N/A
Quote use appropriate, effective	N/A	1	N/A	N/A	N/A	1	N/A	N/A	1	N/A		N/A	3	N/A	N/A	N/A	N/A	N/A	N/A	N/A
Analysis /presentation of negative/deviant cases, alternative explanations	N/A	1	N/A	N/A	N/A	1	N/A	N/A	1	N/A		N/A	3	N/A	N/A	N/A	N/A	N/A	N/A	N/A
Method of reliability check (e.g. triangulation, independent review of data to contest themes)	N/A	1	N/A	N/A	N/A	3	N/A	N/A	1	N/A		N/A	3	N/A	N/A	N/A	N/A	N/A	N/A	N/A
Are findings generalizable to a broader population?	N/A	3	N/A	N/A	N/A	1	N/A	N/A	1	N/A		N/A	2	N/A	N/A	N/A	N/A	N/A	N/A	N/A
Total	12	22	15	9	17	32	12	10	26	15	15	13	47	16	15	15	13	13	10	11
Grading	*	**	*	**	+	+	+	++	++	+	-	++	-	+	+	+	+	+	++	++
*N/A marked under quantitative criteria for qualitative studies and vice versa.																				
RUBRIC																				
++ Low risk of bias: All or almost of the above criteria were fulfilled, and those that were not fulfilled were thought unlikely to alter the conclusions of the study.																				
+ Medium risk of bias: Some of the above criteria were fulfilled, and those not fulfilled were thought unlikely to alter the conclusions of the study.																				
- High risk of bias: Few or no criteria were fulfilled, and the conclusions of the study were thought likely or very likely to alter with their inclusion.																				
